# Thermomechanical Properties of SiC-Filled Polybutylene Succinate Composite Fabricated via Melt Extrusion

**DOI:** 10.3390/polym12020418

**Published:** 2020-02-11

**Authors:** Zelalem Chernet Lule, Eyob Wondu Shiferaw, Jooheon Kim

**Affiliations:** School of Chemical Engineering & Materials Science, Chung-Ang University, Seoul 156-756, Korea; zochernet@gmail.com (Z.C.L.); wendueyoba@gmail.com (E.W.S.)

**Keywords:** polymer matrix composite, extrusion, thermal analysis

## Abstract

Polybutylene succinate (PBS) composites filled with various mass fractions of silicon carbide (SiC) particles were fabricated via slow melt extrusion. The morphological analysis revealed that the fabrication technique assisted in achieving a good adhesion between the PBS and SiC, along with excellent filler dispersion throughout the PBS matrix. The inclusion of 40 wt.% SiC in the PBS composite afforded a 10 °C increase in the thermal degradation temperature and a 160% enhancement in the thermal conductivity relative to the neat PBS. The crystallization temperature also increased with the inclusion of SiC particles, thus making the composites easier to process. Furthermore, the improvement in the Young’s modulus of the PBS composites increased their rigidity and stiffness relative to the neat PBS.

## 1. Introduction

For many years, plastic materials have been manufactured from polymers derived from petrochemical sources. Recently, strict governmental legislations have been implemented to prevent climate change due to the large-scale generation of harmful plastic wastes. Consequently, many researchers are shifting towards fabricating environmentally friendly and biodegradable plastic products [1,2,3]. A large number of widely used biodegradable polymers exist, including polybutylene adipate terephthalate (PBAT), polybutylene succinate (PBS), polylactic acid (PLA), and polycaprolactone (PCL). While the early applications of most of these biodegradable polymers were largely restricted to the medical field [4,5], they are now in greatly increasing demand in a range of fields. In particular, PBS and its composites have recently gained attention because of their good mechanical properties [6,7].

PBS is conventionally prepared via the polycondensation of the monomers succinic acid and 1,4 butanediol, both of which can now be synthesized from renewable sources [8,9,10]. PBS can also be processed using conventional polymer processing techniques such as melt extrusion, casting, compression and injection molding, and hot pressing [11,12,13,14]. Owing to its low rigidity, the commercial application of PBS has been limited to packaging, agriculture, and biomedical sectors [15,16,17]. However, many researches have fabricated filler-reinforced PBS to provide composite materials with enhanced thermomechanical properties. For instance, Gao et al. [18] studied the thermal and mechanical properties of a PBS–magnesium hydroxide sulfate hydrate whisker (UMHSH) composite to achieve significant improvements in the tensile strength, Young’s modulus, and flexural strength. In previous work [19], the present research group has also investigated the thermomechanical properties of an Al_2_O_3_-filled PBS composite fabricated via melt extrusion. These composites exhibited a superior thermal conductivity and storage modulus compared with the neat PBS.

Silicon carbide (SiC) is a ceramic filler often used as a reinforcement in polymer composites owing to its outstanding strength, excellent hardness, and high thermal conductivity [20,21]. Previously, many polymers have been reinforced with SiC to improve their thermomechanical properties. For instance, Mdletshe et al. [22] used mold extrusion to fabricate a PCL composite filled with SiC particles. The incorporation of SiC influenced the thermal stability and the viscoelasticity of the PCL/SiC composites. Liu et al. [23] stated that the thermal conductivity of SiC-filled PLA composites increased with increasing the mass fraction of the filler. Similarly, Liao et al. [24] fabricated a high-density polyethylene (HDPE) composite with improved crystallinity and tensile strength by the addition of various quantities of SiC particles.

To the best of the present authors’ knowledge, studies on the thermomechanical behavior of PBS–SiC composites have not been previously reported. In the present work, SiC-filled PBS composites were fabricated via melt extrusion at a low screw rotation speed designed to enhance the dispersion of the SiC particles throughout the matrix and thus inhibit the formation of aggregates that would otherwise compromise the thermal and mechanical properties of the composites [25,26]. The thermomechanical properties of the fabricated composite samples were characterized by differential scanning calorimetry (DSC), thermogravimetric analysis (TGA), universal testing machine (UTM), and dynamic mechanical analysis (DMA). The results are thoroughly discussed in the following sections and indicate that the PBS–SiC composites generally exhibit an excellent enhancement in thermomechanical properties relative to those of the neat PBS.

## 2. Experimental

PBS composites filled with various loading fractions of SiC (average size: 400 mesh particles) were fabricated via slow melt extrusion followed by compression molding. Prior to the fabrication process, the SiC particles (Sigma-Aldrich, Seoul, Korea) and PBS (Gio-Soltech Co. Ltd., Gangwon-do, Korea) were pre-dried at 70 °C for 24 h in order to remove surface moisture, whichmight otherwise lead to the formation of cracks and voids in the composite system. The pre-dried SiC particles and PBS were mechanically mixed and directly fed into the twin screw extruder (Bau Tech, model BA-11, Seoul, Korea), with an L/D ratio of 40, operating at a screw rotation speed of 30–50 rpm and temperature of 150 °C. The extrudates were immediately cooled and pelletized into small pellets. The specimens for characterization were fabricated from the pellets of the corresponding PBS composites by compression molding (model BA-915, Bau Tech, Seoul, Korea) and were designated as PBS-X, where X represents the loading percentage of the SiC particles.

### Characterization

The morphologies of the freeze-fractured cross-sectional samples of the neat PBS and PBS–SiC composites were examined by field-emission scanning electron microscopy (FE-SEM; Sigma, Carl Zeiss, Oberkochen, Germany) after sputtering the samples with platinum to provide conductivity. The thermal stabilities of the neat PBS and its composites were examined by thermogravimetric analysis (TGA; TGA-2050, TA Instruments, New Castle, DE, USA), which involved heating a small amount of the sample from room temperature to 600 °C at a heating rate of 20 °C·min^−1^ in a nitrogen atmosphere.

The melting and crystallization behaviors of the PBS composites were examined by differential scanning calorimetry (DSC; DSC-Evo, KEP Tech., Mougins, France) stabilized in a nitrogen atmosphere. The analysis was performed by two cycles of heating from room temperature to 160 °C at a heating rate of 10 °C min^−1^. The thermal properties obtained from the second heating cycle were then used to calculate the level of crystallinity (*X_c_*).

The thermal conductivity of the PBS composites was calculated from Equation (1). The thermal diffusivities (δ) of the sample discs (25 mm diameter) were determined using laser flash analysis (Hyper-Flash, LFA-467, Netzsch Instruments Co., Selb, Germany). The density ($\rho_{comp}$) and heat capacity ($C_{p}$) were determined using the water displacement method and differential scanning calorimetry (DSC, DSC-Evo, KEP Tech., Mougins, France), respectively, at room temperature.

(1) $$\kappa =\delta *\rho_{comp}*C_{p}$$

A dog-bone-shaped specimen with dimensions of 21 × 4.6 × 3.2 mm was used to measure the mechanical properties of the composites. A universal testing machine (UTM; R&B Corp., Daejeon, Korea), running at a rate of 5 mm/min^−1^ with a 100-kgf load was used to determine the tensile properties of the composites, at room temperature. The viscoelastic properties of the composites were determined in the temperature range of −80 to 105 °C via dynamic mechanical analysis (DMA; Triton Tech., London, UK) at a frequency of 1 Hz.

## 3. Results and Discussion

**Figure 1:**
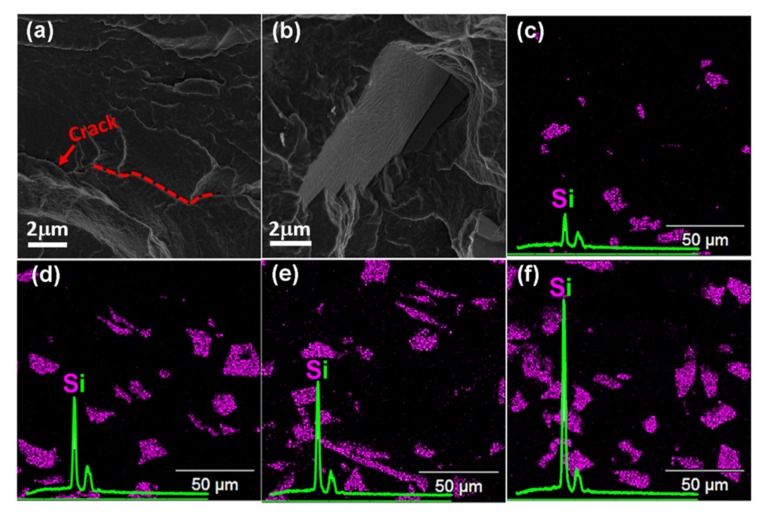
Cross sectional SEM micrographs of the freeze-fractured composites: (**a**) neat polybutylene succinate (PBS) and (**b**) PBS-40. (**c**–**f**) EDXS mapping of the Si atom in the composites: (**c**) PBS-10, (**d**) PBS-20, (**e**) PBS-30, and (**f**) PBS-40.

The PBS composites were prepared using melt extrusion followed by compression molding performed at a similar processing temperature. In addition, the screw rotation speed was controlled so as to provide the feed components with the maximum mixing time, and thus to achieve a good homogeneity with no particle aggregation. The presence of cracks in the freeze-fracture FE-SEM micrograph of neat PBS (Figure 1a) is an indication of its weak mechanical properties. After the addition of SiC, the particles were found to exhibit a good adhesion with the PBS matrix. As shown in Figure 1b, SiC is fully covered and incorporated in the PBS matrix, without showing a clear gap at the interface of the components. Consequently, this excellent interfacial interaction is expected to have a significant effect on the thermomechanical properties of the composites.

In addition, the distribution and loading concentrations of the SiC particles were qualitatively analyzed by Energy-dispersive X-ray spectroscopy (EDXS) mapping of the Si atom. An examination of Figure 1c–f indicates an increase in the Si peak intensity with the increased loading of SiC. The EDSX mapping also confirmed that the control of the extruder rotation speed during the composite fabrication leads to a uniform distribution of the SiC particles throughout the matrix. Hence, with the increase in SiC loading, the formation of numerous PBS–SiC interfaces with a good adhesion leads to an improvement in the dispersion of SiC particles throughout the PBS matrix [27]. As a result, the interfacial interaction of the two components governs the thermomechanical properties of the entire composite system.

**Figure 2:**
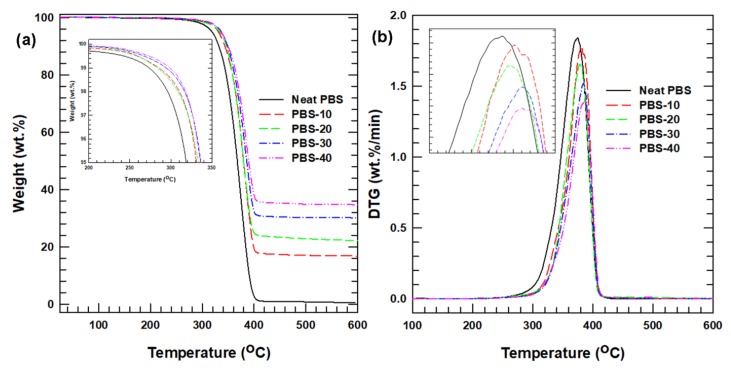
(**a**) TGA thermogram of the neat PBS and the PBS composites; (**b**) derivative of the TGA curve.

The thermal stabilities of the PBS composites were investigated using TGA by heating the samples to above the thermal degradation temperature of PBS. This analysis also helped to verify the successful fabrication of the composites by qualitatively determining the loading percentage of SiC particles after the composites were combusted at a high temperature. The results presented in Figure 2 indicate that the temperature at which degradation begins, i.e., the degradation onset temperature (*T_onset_*), is lower for the neat PBS than for the composites. This demonstrates that the degradation onset temperature of the composites is increased by the incorporation of SiC particles.

In addition, the maximum degradation temperature, indicated by the peak of the DTG curve, shifts to a higher temperature with the addition of SiC. The highest degradation temperature of 350 °C is observed for the PBS-40 sample, which represents a 10 °C improvement relative to the neat PBS. Furthermore, the peak height of the DTG curve decreases as the SiC loading is increased. The peak height of the DTG curve indicates the rate of thermal degradation, i.e., the greater the peak height, the more rapidly the mass is lost. This suggests that the neat PBS degrades much more rapidly than the corresponding composites. In general, the addition of SiC particles improved the thermal stability of the PBS composites by increasing the *T_onset_* and lowering the rate of thermal degradation.

**Figure 3:**
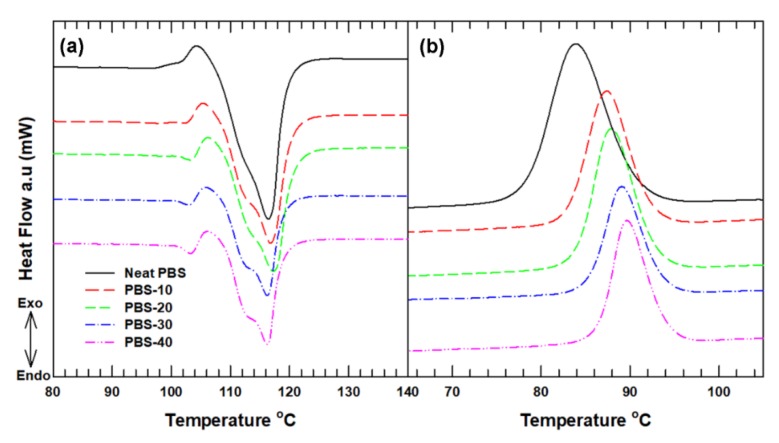
DSC thermogram of the neat PBS and the PBS composites: (**a**) heating (endothermic) peaks and (**b**) cooling (exothermic) peaks.

The melting and crystallization trends of the PBS composites were determined using DSC with two cycles of heating. The overall thermal behavior of the PBS composites is presented in Table 1. The melting behavior was determined from the second heating cycle of the PBS composites (see Figure 3a). It shows that the endothermic melting temperature (*T_m_*) peaks do not show a significant change with the addition of SiC particles in the PBS. However, the melting enthalpy indicated by the area under the endothermic peaks is seen to decrease with the increased loading of SiC. This reduction in the melting enthalpy can be correlated with the dilution effect of the SiC particles within the PBS matrix. In addition, the incorporation of SiC limits the movement of the PBS molecular chain and the released melting enthalpy is reduced, leading to changes in the crystalline properties of the PBS composites [28]. The degrees of crystallinity of the semi crystalline PBS composites were calculated using Equation (2):

(2) $$X_{C}\left(\%\right)=\frac{∆H_{m}}{∆H_{m}^{O}\times \;W_{PBS}}\;\times 100$$

where $X_{C}$ is the degree of crystallinity of PBS, $∆H_{m}^{O}$ is the enthalpy of fusion of 100% crystalline PBS (200 J g^−1^) [29], Δ*H*_m_ is the melting enthalpy of the PBS composites, and $W_{PBS}$ is the weight fraction of PBS in the composites. The degree of crystallinity of the PBS composites is lower than that of the neat PBS. Even though the drop in $X_{C}$ is inconsistent with the SiC loading, it further confirms that the filler particles hindered the migration and diffusion of PBS chains, lowering the degree of crystallinity.

In contrast to the melting temperature, the crystallization temperature (*T_c_*) recorded from the exothermic peaks of the cooling cycles displayed an increase with the inclusion of SiC particles, which indicated that SiC particles act as a nucleating agent of PBS, thus helping to close the temperature gap between the crystallization and melting temperature during the cooling process while the composite is being used in various fields of application. When this temperature gap is close enough, it helps the polymer composite to crystalize naturally, without using coolant or quenching processes that can deteriorate the mechanical properties of the composites. In addition, the crystallization time (*t_c_*) given by the time difference between the onset crystallization temperatures, where the crystallization process starts, and the offset crystallization temperature, where the crystallization process is fully completed, decreased significantly with the addition of the filler loading. This decrease in crystallization time allows the composites to form crystals readily at an early stage of crystallization. In general, these thermal behaviors relating to polymer chain relaxation are expected to influence the other thermomechanical properties of the composites.

**Figure 4:**
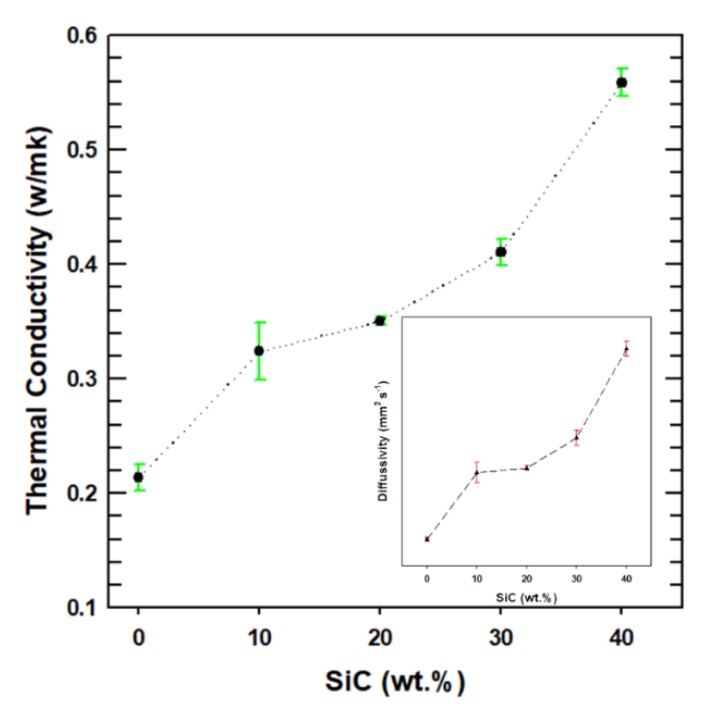
Thermal conductivity of the neat PBS and PBS composites.

The thermal conductivity of the polymer composites is an important property that determines the performance and application of the materials. There are various factors that affect the thermal conductivity of a composite, such as the size and distribution of fillers throughout the polymer matrix, the loading fraction of thermally conductive fillers, and the interfacial adhesion between the filler and matrix. The thermal conductivities of the PBS composites were calculated using Equation (1) and are presented in Figure 4. The conductivity is seen to increase with the increased loading of SiC, with a particularly large increase between the PBS-30 and PBS-40 composites. The thermal conductivity of the PBS-40 composite is 0.56 W/mK, which represents a 160% enhancement relative to that of the neat PBS. Hence, the interfacial adhesion and filler dispersion contributes to the improved thermal conductivity of the PB-40 composite by creating a good thermal transport pathway for the heat to be dissipated throughout the composite system.

**Figure 5:**
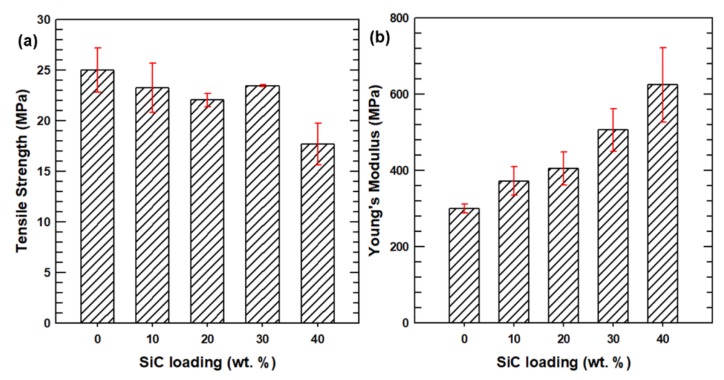
Tensile properties of the neat PBS and PBS composites: (**a**) tensile strength and (**b**) Young’s modulus.

The average tensile test results for each PBS composite are presented in Figure 5. The tensile strength is seen to decrease slightly with the addition of SiC particles up to 30 wt.%. The PBS-40 composite exhibits the lowest tensile strength of 17.7 Mpa, which is around 8 Mpa lower than that of the neat PBS. This result was expected because the inclusion of SiC particles lowers the stress transfer at the interface when tensile deformation is applied to the composites, which, in turn, affects the PBS chain relaxation. The resulting concentration of the stress at the interface makes the polymer chains become rigid and then generates cracks during deformation, leading to a decrease in tensile strength. By contrast, the Young’s modulus of the PBS composites is seen to increase uniformly with the addition of SiC particles. Ceramic fillers such as SiC are known to have a high resistance to load deformation, which demonstrates their high Young’s modulus and stiffness. Increasing the loading fraction of the fillers therefore increases the Young’s modulus of the composite system. As a result, the PBS-40 composite exhibited an average Young’s modulus of 685 Mpa, which is over twice that of the neat PBS. In general, these PBS composites can be used for applications that require moderate strength with a high stiffness.

**Figure 6:**
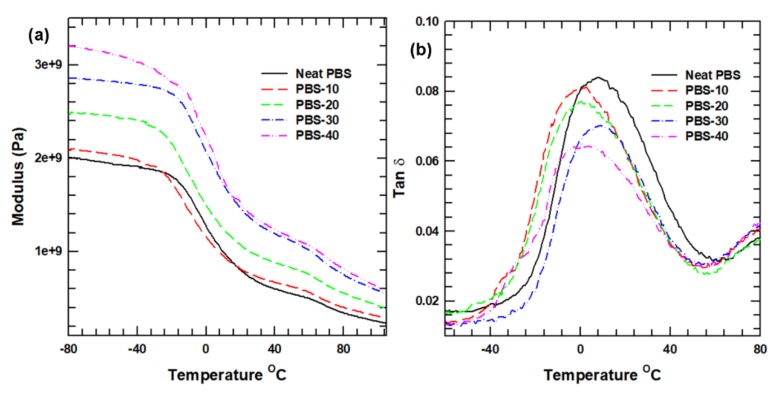
Viscoelastic properties of the neat PBS and the PBS composites: (**a**) storage modulus and (**b**) loss factor.

The viscoelastic properties (i.e., the storage modulus and tan *δ*) of the PBS composites were investigated using DMA, and the results are presented in Figure 6. In full agreement with the Young’s modulus results, the storage modulus of the PBS composites displays an initial increase with the addition of SiC fillers. The PBS-40 composite displays a 60% improvement in the storage modulus, relative to that of the neat PBS. However, as the temperature increases, the storage modulus begins to decrease drastically, due to the polymer chain relaxation that occurs at the glass transition temperature. The polymer chain relaxation is usually represented by the tan δ curve (Figure 6b). The height of the tan *δ* peaks of the PBS composites, which indicates the mobility of the amorphous region of the matrix, is seen to decrease with the addition of SiC particles, thus indicating an increased elasticity and increased potential to store load rather than dissipating it. Similarly, the addition of SiC particles creates restrictions against the molecular motion of the polymer, thus increasing the stiffness and rigidity of the PBS composites relative to the neat PBS.

## 4. Conclusions

The commercial demand for biodegradable polymer-based plastic materials has significantly increased because of climate change and the excessive generation of wastes associated with the use of petrochemical-based polymer materials. In the present work, silicon carbide (SiC)-filled PBS composites were synthesized by a slow melt extrusion technique. The good adhesion of the SiC particles to the PBS matrix, and their excellent dispersion throughout the matrix were indicated by FE-SEM analysis. Consequently, the thermomechanical properties of the PBS composites were significantly influenced by the inclusion of SiC. The PBS composites exhibited enhanced thermal stabilities relative to the neat PBS, and the thermal conductivity was increased by 160% with the addition of 40 wt.% SiC. The Young’s modulus of the PBS composites also showed a good improvement with the addition of SiC to the PBS. In general, the SiC particles significantly influenced the thermomechanical properties of the PBS composites.

## Figures and Tables

**Table 1:** Thermal behavior of the neat PBS and its composites. *T_m_*—melting temperature; *T_c_*—crystallization temperature; *t_c_*—crystallization time; Δ*H*_m_—melting enthalpy of the PBS composites; *X_c_*—level of crystallinity.

Sample	*T_m_* (°C)	*T_c_* (°C)	*t_c_* (s)	Δ*H_m_* (J g^−1^)	*X_c_* (%)
Neat PBS	116.7	83.9	158.3	86.1	43.0
PBS-10	116.7	87.4	99.9	69.6	38.7
PBS-20	117.2	87.9	94.7	58.9	36.8
PBS-30	116.2	89.0	76.9	54.6	39.0
PBS-40	116.2	89.7	76.4	49.6	41.3
